# Challenges of using external data in clinical trials- an illustration in patients with COVID-19

**DOI:** 10.1186/s12874-022-01769-5

**Published:** 2022-12-15

**Authors:** Sylvie Chevret, Jean-François Timsit, Lucie Biard

**Affiliations:** 1 Department of Biostatistics, Hôpital Saint-Louis, Paris, France; 2 ECSTRRA Team, INSERM U1153,Université de Paris, 75010 Paris, France; 3 Medical and infectious diseases ICU, Hôpital Bichat-Claude-Bernard, 75018 Paris, France

**Keywords:** Borrowing, Clinical Trials, External data, Indirect comparison

## Abstract

**Background:**

To improve the efficiency of clinical trials, leveraging external data on control and/or treatment effects, which is almost always available, appears to be a promising approach.

**Methods:**

We used data from the experimental arm of the Covidicus trial evaluating high-dose dexamethasone in severely ill and mechanically ventilated COVID-19 patients, using published data from the Recovery trial as external data, to estimate the 28-day mortality rate. Primary approaches to deal with external data were applied.

**Results:**

Estimates ranged from 0.241 ignoring the external data up to 0.294 using hierarchical Bayesian models. Some evidence of differences in mortality rates between the Covidicus and Recovery trials were observed, with an matched adjusted odds ratio of death in the Covidicus arm of 0.41 compared to the Recovery arm.

**Conclusions:**

These indirect comparisons appear sensitive to the method used. None of those approaches appear robust enough to overcome randomized clinical trial data.

**Trial registration:**

Covidicus Trial: NCT04344730, First Posted: 14/04/2020; Recovery trial: NCT04381936

## Background

Clinical trials attempting to improve design and analysis to provide more rapid and valid answers, are common. This is particularly true in the setting of rare or paediatric diseases, or in the setting of a new disease, such as COVID-19. Indeed, in the COVID-19 pandemic, many studies have been initiated worldwide to assess the potential effects of various treatments, including antiviral, anti-inflammatory and immunomodulatory therapies. To provide a rapid answer, the use of single-arm clinical trials is becoming increasingly important. In this setting, the “borrowing” of external data, which is commonly available, though underused, appears promising.

However, the ability to leverage existing data differs depending on the type of available data, either limited to the control arm - with the aim of fully replacing the absence of control arm in uncontrolled phase II trials - or with the aim of increasing the amount of information in the current dataset. The first method provides historical control data to fully replace an unobserved control in a single-arm trial [1]. This allows clinical research to be conducted more rapidly with fewer patients and reduced costs compared to a randomized clinical trial, and is particularly useful in settings where the disease or disease subset is rare. This method further addresses an important patient concern, avoiding patients from being exposed to a placebo arm or a standard of care therapy from which little benefit is expected, reducing barriers to recruitment in the trial. Importantly, it should be determined that this method will not compromise scientific evidentiary standards. Recently, such a use of a control arm based on external data has been extended to concurrent real-world data (RWD) and is newly referred to as the “synthetic control arm”, requiring adequate statistical methods to evaluate the comparative efficacy of an intervention [2].

The aim is to increase information by borrowing external data on control and/or treatment effects, which is almost always available. Indeed, many sources of data regarding a particular treatment and population of interest are often available from published data, previous trials or RWD, including cohorts, registries, or electronic medical charts. The US Food and Drug Administration (FDA) and European Medicines Agency (EMA) have recognized these issues and taken several initiatives to allow for these novel approaches to external control data. Many approaches have been proposed for leveraging these external data, to handle the difference (“drift”) between historical and current data. They differ in terms of the type of available data, whether the data are individual patient data (IPD) or aggregated data, and on the primary or secondary outcomes, with the use of only external control data being the most common.

The process of borrowing external data uses various Bayesian approaches that primarily differ in the assumptions regarding the relevance and exchangeability of the external data with the current trial [3–5]. They typically use power priors, down-weighting controls using fixed weights [3, 6], or more complex meta-analytic priors such as normalized power prior [7], commensurate priors [4], robust meta analytic-predictive priors (MAP) [8, 9], and supervised methods that manually adjust the informativeness of the prior based on measures of conflict between the prior information and the new trial data, assessed at the time of the analysis. Such a variety of proposed approaches was illustrated in a review that identified 58 Bayesian and 44 frequentist methods for incorporating historical data into a contemporary trial based on 52 articles [10].

In addition to those Bayesian methods, there is room for frequentist approaches that take advantage of the functional differences between external and current populations. Indeed, frequentist propensity score approaches are widely used to utilize RWD in combination with concurrent experimental data in clinical trials, to eliminate or reduce the potential bias in estimated effects obtained from nonrandomized comparative studies. Notably, matched-adjusted indirect comparisons (MAIC) use a propensity–score approach to adjust for baseline differences in those populations, when only aggregated data are available from the historical data [11].

However, little attention has been devoted to the practical use and implementation of methods incorporating external information, and how to pick one among them, notably according to their underlying assumptions. We illustrated the use of different statistical methods that aim at incorporating external information, based on a real-life trial addressing the effect of dexamethasone in severe COVID-19 patients. Data from one randomized clinical trial (Covidicus) conducted from April, 10, 2020, to January, 2021 (NCT04344730) was used as the current trial data. We focused on the subset of mechanically ventilated patients, given the marked reported benefit in this subset over usual care, from the previously published Recovery trial (NCT04381936) [12]. We borrowed results from the Recovery trial [12] to estimate the 28-day mortality rate of the dexamethasone arm from the Covidicus trial [13] as the parameter of interest. We thus only considered the two treatment arms of these trials, mimicking two single-arm trials. This was in line with the growing use of uncontrolled phase II trials in clinical settings. This was further motivated by the fact that the results of the Covidicus trial were not published at the time we designed this study, to ensure maintenance of blindness. Moreover, focusing on the death rate allows us to provide an easy and direct understanding of the models, rather than assessing relative measures of efficacy.

## Methods

### Covidicus Trial

The trial was scheduled, in March 2020, to assess the impact of high-dose dexamethasone on overall mortality in patients admitted to intensive care units (ICUs) for severe COVID-19 infection. The primary endpoint was 60-day mortality, and 28-day mortality was one of the secondary endpoints.

Patient enrolment occurred from April, 10, 2020 to January, 25, 2021, with a total of 550 enrolled patients. As an illustrative example of using external data for uncontrolled designs, we only considered the experimental arm of the Covidicus trial, i.e., the high-dose dexamethasone, allocated to 106 mechanically ventilated patients.,

We thus proposed to incorporate the Recovery results of the dexamethasone arm [12], considering both aimed to estimate the benefit of dexamethasone in MV patients with COVID-19. A comparison of the main trial characteristics is summarized in Table 1, to assess the potential sources of heterogeneity across trial populations as reported by Pocock [1]. Compared to the Recovery trial patients, Covidicus patients were older, and more likely to be men with comorbidities.

**Table 1:** Comparison of the Covidicus and Recovery trials based on the Pocock criteria of population homogeneity

Criteria	Covidicus	Recovery
Treatment	High DXM	Low DXM
Eligibility	Age>18	No age limit
Treatment evaluation	Death at day 28 and 60	Death at day 28
Characteristics		
Age (years, mean ± standard deviation)	66 ± 11	59 ± 13
Male sex	80%	73%
Comorbities	80%	49%
Organisation	France	UK
Selection	April, 20- January, 21	March-June, 20

### Estimation of the 28-day death rate of the dexamethasone arm of the current trial

First, we borrowed Recovery data (referred to as “historical data” hereafter) to estimate the 28-day death rate of the dexamethasone arm in the Covidicus trial (the “current” trial hereafter).

#### Bayesian Standard Model

Let $\theta$, define the parameter of interest, namely the 28-day mortality rate, and let $\pi (\theta )$, be the prior distribution for $\theta$. The prior represents all information and prior beliefs regarding the parameter of interest before the trial onset. Based on the Bayes theorem, it can be combined with the likelihood function $L(D|\theta )$ into the posterior distribution of $\theta$, $\pi (\theta |D) \propto L(D|\theta ) \pi (\theta )$ where $D=\{y, n\}$ is the current trial data, with *y*, the number of deaths and *n* the sample size.

Using Bayesian models with priors represents a natural approach for incorporating historical data for estimates using single arm evidence [14].

To borrow external data, $D_h=\{y_h,n_h\}$, we first assume clinical homogeneity of the past and current populations, i.e., no main variability among trials in the participants’ characteristics, as well as in the intervention characteristics that may result in any heterogeneity in treatment effect. This could rely on a lack of information regarding the predictive factors of treatment effect, or on the failure of detecting any predictive factors of treatment effect in the population of interest. In these settings, the idea is to use $\pi (\theta |D_h)$ as the prior for $\theta$, that is, some distribution including the external data in some sense.

First, most Bayesian methods use existing data in the source population to create an informative prior distribution for the future clinical trial. The simplest way is to use the posterior distribution of the historical data $D_h$, as the prior for the current data:

1 $$\begin{aligned} \pi (\theta |D_h)\propto L(D_h|\theta ) \pi (\theta ) \end{aligned}$$

where $\pi (\theta )$ is the prior for the historical data.

Our information on $\theta$ is then actualized, resulting in the following:

2 $$\begin{aligned} \pi (\theta |D,D_h) \propto L(D|\theta ) L(D_h|\theta ) \pi (\theta ) \end{aligned}$$

where $L(D_h|\theta )$ and $L(D|\theta )$ are the likelihood functions of the external and the current data, respectively. In doing so, equal weights are given to past and current data, due to the underlying assumption of strictly homogeneous populations. This is likely to be true when several trials addressing the same question have been conducted in the same population by different teams due to a new epidemics such as the COVID-19. This could also rely on rare populations with no available or demonstrated knowledge on which factors may affect the likelihood of patient response to treatment.

Otherwise, prior parameters could be based upon eliciting observable quantities of the past data such as the mode and percentiles, as widely reported in the literature [15]. Indeed, solving for the parameters of a Beta distribution under quantile constrains, is numerically possible, where the only difficulty comes from the Beta function involved as a normalizing constant of the Beta distribution [16].

To understand the relative weights of external and current data, one can formulate Bayes learning in terms of linear shrinkage. Given $\theta \in [0,1]$, a common prior $\pi (\theta )$ is the *Beta*(*a*, *b*) family, with $a>0,b>0$ acting as pseudo-counts that influence both the posterior mean and the posterior variance, in exactly the same way as conventional data. Indeed, due to its conjugacy with the binomial likelihood, posterior density is easily obtained by updating its parameters, as follows, $Beta(a+y_h+y,b+n_h+n-y_h-y)$. Thus, the posterior mean $E(\theta |D,D_h)= (1-\lambda ) E(\theta |D_h) + \lambda \hat{\theta }$ is a weighted average of the prior mean $E(\theta |D_h)$ and the ML estimate $\hat{\theta }$ of $\theta$, where $\lambda =\frac{m}{m+n} \in [0,1]$ is the shrinkage intensity, and $m=a+b+n_h$. Thus, when the sample size of the external data $n_h$ is widely above that of the current data *n*, it is likely that most of the information regarding the parameter will rely on those past data.

To erase the weight of external information compared to current data, for instance due to differences in sample sizes or in data sources (RWD vs trial data), the shrinkage intensity can be decreased. This can be done by modifying the prior parameters. Indeed, the parameter of the Beta prior (*a* and *b*) act as pseudo-counts that influence both the posterior mean and the posterior variance, exactly in the same way as conventional data. The effective sample size (ESS), $m=a+b$, behaves like an implicit sample size connected with prior information. Thus, one can down-weight the external data by modifying the prior parameters. For instance, one may use the mean $\overline{y_h}$ of the external data to define the prior mean of the parameter, then defining some amount of information *m* that one wish to affect to these data. This will define the prior parameters, $a=\overline{y_h} \times m$, and $b=m-a$, for the current data, from which the posterior density will be derived $Beta(\overline{y_h} \times m+y,m-a+n-y)$.

Nevertheless, converting prior knowledge into a prior is almost never an exact process. Whatever the prior, one should assess its effect on the posterior, and by contrasting the posterior from one resulting from a non-informative prior.

**Fig. 1:**
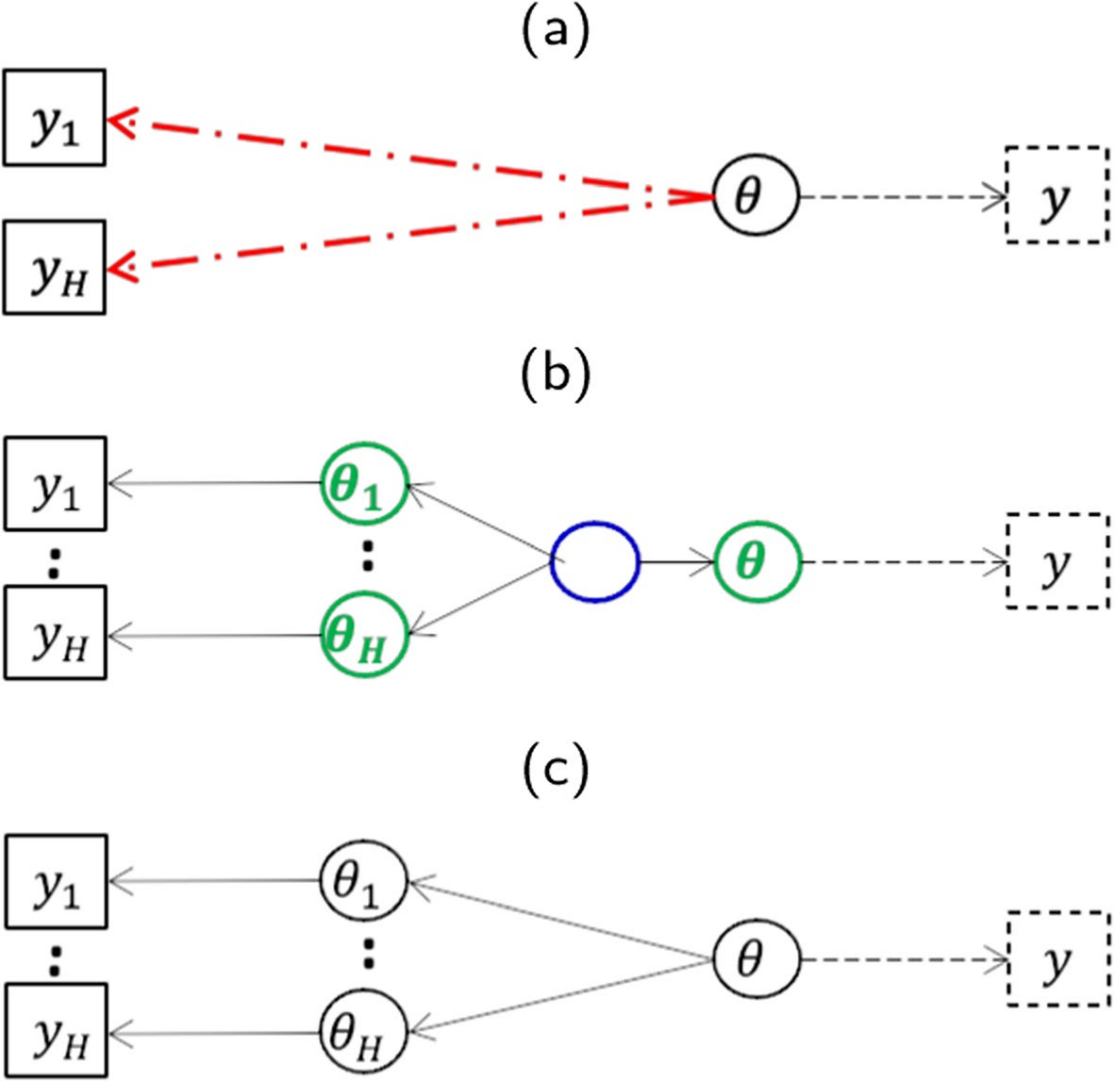
Modelling the data to handle prior and current data heterogeneity. Upper panel (a) refers to the power prior models, middle panel (b) refers to the hierarchical Bayesian models, and lower panel (c) refers to the potential bias approach of Pocock

All these Bayesian analyses are equivalent to pooling data, assuming no main heterogeneity between the external and current data. When populations are not so identical, that is, when faced with any doubt about comparability, or suspicion of heterogeneity, several approaches could be used, that differ in their principle: by discounting, erasing or shifting the prior data (Fig. 1).

#### Power Prior Models

If there is any doubt about comparability or any suspicions of heterogeneity between the two populations, the first method is to discount, wherein down-weighting the external data occurs. Still assuming a similar parameter $\theta$ across the datasets, power priors [3] consist in raising the likelihood of the historical data to an exponent $a_0$ representing the commensurable degree between the historical and new trial data. The previous prior (in Eqs. 1 and 2) is switched to the following:

3 $$\begin{aligned} \pi (\theta |D_h,a_0)=L(D_h|\theta )^{a_0} \pi (\theta ) \end{aligned}$$

where $a_0 \in [0,1]$ controls the weight given to the historical data in the posterior distribution. This posterior is indeed used as the prior for the new study.

Thus, the power prior calculates a posterior distribution from a prior and the weighted likelihood of the previous data. One can interpret the posterior as including $a_0 \times n_0$ patients from the external study in addition to those from the current study.

The major criticism of the power prior has been the difficulty in choosing the weight parameter, $a_0$. To choose the value of $a_0$, the easiest solution could be to use a hierarchical power prior by specifying a proper prior distribution for $a_0$. Random $a_0 \sim \pi (a_0|\gamma _0)$ allows more flexibility in weighting the historical data:

4 $$\begin{aligned} \pi (\theta |D_h) = \int _0^{1} \pi (\theta ,a_0|D_h) da_0 \propto \int _0^{1} L(D_h|\theta )^{a_0} \pi (a_0|\gamma _0) \pi (\theta ) da_0 \end{aligned}$$

where $\gamma _0$ is some specified hyperparameter vector, and $\pi (\theta ,a_0|D_h)$ is the joint prior distribution of $\theta$ and $a_0$.

However, taking $a_0$ fixed and doing several sensitivity analyses for different values of $a_0$ has been reported computationally feasible and easier to interpret than taking $a_0$ random [17]. To avoid prior misspecification, several guidance approaches have been proposed such as the Empirical Bayes estimate of the prior weight parameter based on the data by marginal likelihood [18].

#### Bayesian hierarchical models

Borrowing from similar, previously conducted trials accounting for variations in study design, baseline characteristics, and standard-of-care improvement, using hierarchical Bayesian methods has also been proposed [19, 20].

Contrary to the previous approach, the parameter of interest of the current and historical data, namely $\theta$ and $\theta _h$ are considered randomly drawn from a common distribution, whose mean and variance are inferred from the data. In other words, this approach is based on a mixed effect model where $\theta$ and $\theta _h$ follow a similar $Beta(a_B,b_B)$ distribution where $a_B$ and $b_B$ are assigned uninformative priors.

#### Bias approach

Another type of hierarchical model that assumes historical response rate $\theta _h$ is a nonsystematically biased version of the current response rate $\theta$. Rather than assuming that they are exchangeable such as in previous random effects model, Pocock [1] assumes that the parameter from the external data is biased: $\theta _h=\theta + \delta _h$.

Three options can be considered to define the bias, $\delta _h$. The bias could be (i) fixed, (ii) centered on zero, $\delta _h \sim N(0,\sigma _{\delta _h}^2)$, or (iii) not $\delta _h \sim N(\mu ,\sigma _{\delta _h}^2)$, where $\sigma _{\delta _h}^2$ is the interstudy variance set fixed or estimated from the data.

#### Comparison of models

The effective sample size (ESS), that has been proposed to quantify the amount of prior information as a measure of prior influence, [21], acts as an important comparator across models, the effective number of either fictive or historical patients borrowed, as it pertains to the treatment effect between the current data and the concurrent data [22]. For a *Beta*(*a*, *b*) prior, it is defined as $m=a+b$.

### Comparison of the 28-day mortality rate in the dexamethasone arm between historical and current trials

We first assumed that any dose used by the trial authors, were relevant, that is, there was an underlying assumption that the lowest dose will have a similar (even if not exact) effect as the highest dose (and vice versa). That is often a fair assumption used in meta-analyses, where all the intervention arms are combined into a single ’treatment’ arm versus the comparator. Nevertheless, on the other hand, there can be a heterogeneity in true benefit or harm from the different doses. Thus, we then aimed to compare the effect of the two dosages; this was indeed the aim of the Covidicus trial. We only considered how to provide some answer to that question, based on the data from two (hypothetic) single-arm trials. Moreover, given the obvious differences across historical and current trials, notably in the treatment doses, we aimed to compare the 28-day mortality rate between the low (in the Recovery trial) and high (in the Covidicus trial) dexamethasone arms, handling these potential confounders. This will allow us to exemplify frequentist-based approaches.

#### Direct unadjusted comparison of mortality rates

When individual patient data are available or can be reconstructed such as from response/death rates, frequentist methods can synthesize data from different populations using either a joint model or a weighted test statistic.

#### Direct adjusted comparison of mortality rates

To handle observed differences in confounders across groups, propensity score (PS) approaches have been proposed, which mostly used to infer treatment effect comparisons from observational studies. They require individual patient data on potential confounders and treatment modifiers, not only on outcomes, for both treatment groups to be compared.

#### Matched–adjusted indirect comparisons

When only aggregated historical controls are available, matching-adjusted indirect comparisons (MAIC) propose weighting the IPD to bring them more in line with the historical controls similar to surveys [11, 23]. This method is widely used when no head-to-head comparison is available, but individual patient data are only available for one comparison/drug. It is a propensity score (PS) weighting approach, where patients in trials with IPD are weighted such that their weighted mean baseline characteristics match those reported for the trials without IPD but with only published aggregate data. The PS model is estimated using the generalized method of moments to handle the data structure. After matching, outcomes can be compared across balanced trial populations, using weighted statistical tests that incorporate the weights developed in the matching process. This can be done using a simple weighted average of the outcome in the Covidicus trial, or using a weighted linear regression without covariates. The advantage of the latter approach is that so-called “sandwich” estimators for the variance can be used, which will yield correct confidence intervals, accounting for the fact that the weights themselves are estimated.

The R library MAIC (https://github.com/heorltd/maic) was used.

## Results

In the Covidicus trial, 106 patients who were mechanically ventilated at baseline were enrolled and allocated to the high dexamethasone arm, including 25 (23.58%, 95% confidence interval, 95%CI, 15.88 to 32.82) who died within the first 28 days of randomization. Of the 324 patients who were randomly allocated to the dexamethasone arm in the Recovery trial, 95 (29.32%, 95%CI, 24.42 to 34.60) died.

### Estimating the 28-day mortality rate in response to Dexamethasone treatment

We first exemplified the use of Bayesian methods. We considered noninformative Beta(1,1) prior information as the reference for the Covidicus 28-day mortality rate. Ignoring the external data, given an effective sample size $m=2$ and the shrinkage intensity $\lambda =0.018$, the posterior mean of the 28-day mortality rate was $E(\theta |data)=0.2407$ (Table 2).

**Table 2:** Posterior Bayesian estimates of the 28-day mortality rate in the Covidicus trial using the simplest Bayes models according to the approach. CrI: Credibility Interval

Approach	Prior for$\theta$	Parameter	Mean	95%CrI interval	
**Ignoring external data**					
Simple Bayes	Beta(1,1)	$\theta$	0.2407	0.1653	0.3253
**Incorporating external data**					
Combining data	Beta(96,230)	$\theta$	0.2801	0.2388	0.3233
Modifying the prior					
- Based on quantiles	Beta(77,185)	$\theta$	0.2771	0.2327	0.3239
- Based on shrinkage intensity,$m=10$	Beta(2.9,7.1)	$\theta$	0.2408	0.0711	0.5927
- Based on shrinkage intensity,$m=100$	Beta(29.3,70.7)	$\theta$	0.2637	0.2086	0.3856
Fixed Power Priors, EB estimate		$\theta$	0.2725	0.2208	0.3240
Random Power Priors	Beta(1,1)	$\theta$	0.2479	0.1770	0.3176
		$a_0 \sim$Beta(1,1)	0.1955	0.0001	0.6212
		$\theta$	0.2390	0.1675	0.3192
		$a_0 \sim$Beta(1/2,1/2)	0.1072	0.0001	0.4887
		$\theta$	0.2473	0.1697	0.3160
		$a_0 \sim$Exp(100)	0.1930	0.0001	0.6232
Hierarchical Bayesian Models					
	Beta(1,1)	$\theta$	0.2944	0.2480	0.3457
	Beta(1/2,1/2)	$\theta$	0.2934	0.2450	0.3440
Pocock’s bias Approach	*N*(0, 0.03)	$\delta _h$	-0.00035	-0.0629	0.0549
		$\theta$	0.2770	0.2355	0.3194
	*N*(0.07, 0.03)	$\delta _h$	-0.0697	0.00706	0.1249
		$\theta$	0.2770	0.2365	0.3194
	$N(-0.07,0.03)$	$\delta _h$	-0.0703	-0.13296	-0.0151
		$\theta$	0.2770	0.2365	0.3194

To incorporate external information from the Recovery trial, the choice of the Bayesian model depends on the assumptions related to the heterogeneity between populations.

#### Ignoring heterogeneity

We first pooled all observed data from the two trials, assuming homogeneous populations. One may indeed suppose that there were large uncertainties on prognostic and predictive features in severe, mechanically ventilated, COVID-19 patients, at the time of onset of the COVID-19 outbreak.

As naturally provided by the Bayesian inference, the posterior distribution $\pi (\theta | y, y_h,a)$ could be used as a prior for the current data. Thus, the prior information was first based on the observed $y_0=95$ deaths among the $n_0=324$ patients. Combining past and current data with a non-informative prior beta(1,1) resulted in a posterior mean of 28-day mortality of 0.28 (95% credibility interval, 95%CrI, 0.239 to 0.323).

**Fig. 2:**
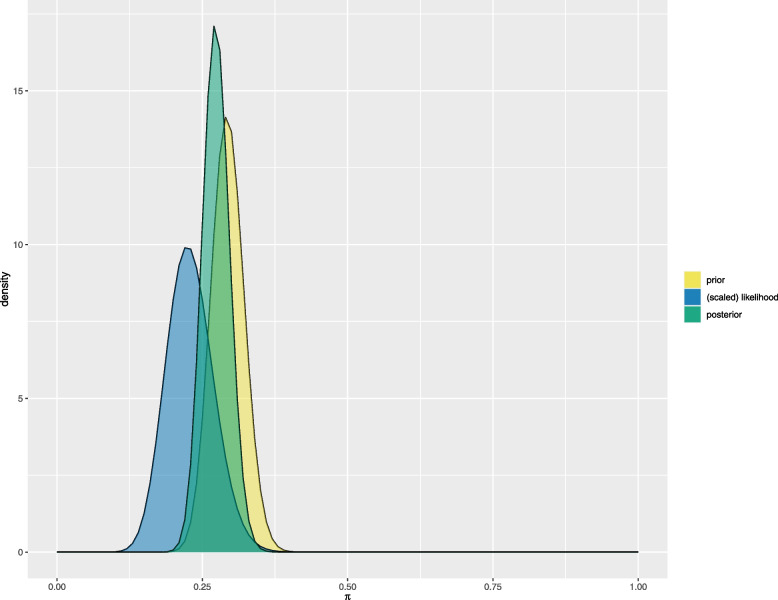
Bayesian analysis of the Covidicus trial data incorporating Recovery trial data assuming homogeneity of data

Otherwise, rather than incorporating roughly the external sample data, one may define the Beta prior from the estimates of the death rate from the Recovery external data. We selected the shape parameters of the Beta density that matched knowledge of two quantiles of the distribution, based on the bounds of the exact binomial 95% confidence interval (0.24, 0.35) of the ML estimate of the 28-day mortality rate from the reported trial data. This resulted in using as the prior for the Covidicus trial the Beta(77,185) prior, with prior mean of 0.29, similar to the ML estimated from the Recovery trial. Figure 2 shows the prior, likelihood and posterior densities of the 28-day mortality. The effective sample size became $m=262$, while the sample size was $n=106$, resulting in a shrinkage intensity at $\lambda =0.71$. The posterior mean of $\theta$ became 0.2771, while when pooling all data from the two trials, regardless of their source, reached 0.2801, which is closest to the ML estimate of 0.29 from the Recovery trial (Table 2).

**Fig. 3:**
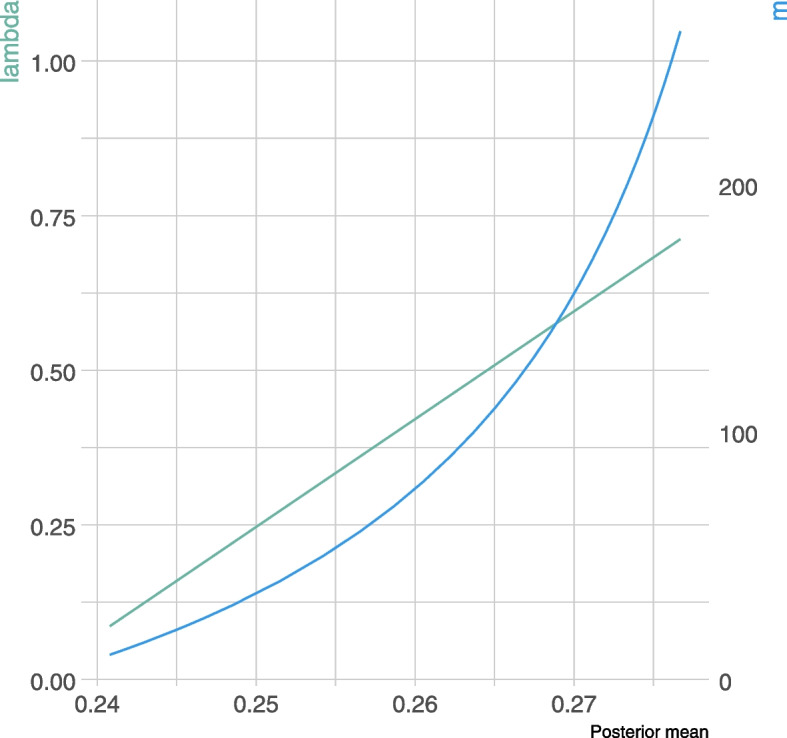
Estimation of 28-day mortality rate using empirical Bayes models according to the shrinkage intensity, $\lambda$ and the effective sample size, *m*

However, by ignoring any heterogeneity across samples, it is likely that the weight attributed to the prior overpasses that of the current sample, due to imbalance in sample sizes. One simplest way to down-weight the past data (and erase the impact of its large sample size compared to that of the current sample), consists in modifying the prior parameters so that the shrinkage intensity is decreased. Thus, we reran the posterior estimates of the death rate, using the prior Recovery mean, but varying the effective sample size *m* from 2 to 262, that is, the shrinkage intensity from 1.8% up to 71.2%. As expected, the posterior mean increased with the shrinkage intensity, that is to the relative weight of the external data (Fig. 3).

However, referencing the Pocock criteria [1], there appears to be some heterogeneity between the trials that should be considered (Table 1). We acknowledged that some further findings have outlined the prognostic and predictive value of age and comorbidities, so that heterogeneity across trials had to be taken into account.

#### Handling heterogeneity through down-weighted power priors

External data were first discounted, and down-weighted, relatively to the current data, using power priors.

**Fig. 4:**
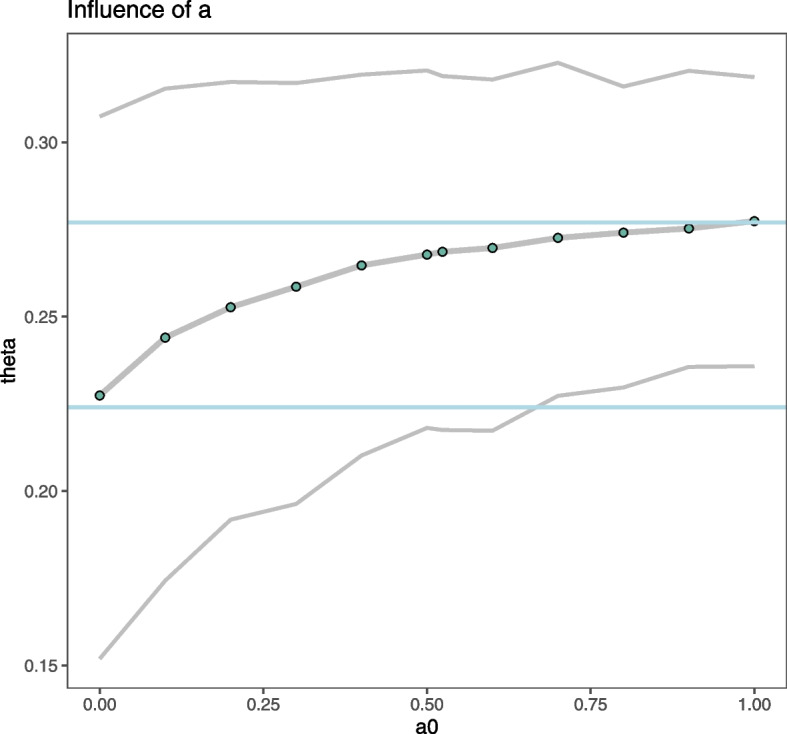
Estimation of 28-day mortality rate using power prior models according to the power model parameter, $a_0$. The blue lines indicate the position of the standard Bayes estimate from only the Covidicus trial data (when $a_0=0$) and that of ignoring heterogeneity in trial data (when $a_0=1$)

One of the most difficult and elusive issues in the use of the power prior is the choice of power prior parameter $a_0$. First, it was considered fixed, ranging from 0 to 1, while the prior was set as Beta(1,1). Results are shown in Fig. 4. As expected, estimates ranged from 0.2407 (95%CrI, 0.1652 to 0.3351), that is the posterior estimate reached ignoring the Recovery trial data up to 0.2801 (95%CrI, 0.239 to 0.323), which was obtained when roughly incorporating the external data but ignoring any heterogeneity between data sets.

We last applied the Empirical Bayes estimate proposed by [18]; a value of $a_0=0.52$ was found. In this case it is equivalent to 170 additional patients (rather than 324). This resulted in a posterior mean of death rate at 0.2725 (95% CrI, 0.2208 to 0.3240).

Second, we considered a random power prior parameter, drawn from either the Beta or exponential families. The posterior estimate of $\theta$ lies in the previous range of estimates, with a poor influence of prior parameter density (Table 2).

#### Handling heterogeneity through hierarchical models

Another and the most common way of handling heterogeneity (as performed in meta-analysis) is to use a hierarchical model, where the prior parameters *a* and *b* were both drawn from a Beta(1,1) distribution. This resulted in a posterior mean of $\theta$ of 0.2944 (95% CrI, 0.2480 to 0.3457). Using the Jeffrey prior for $\theta$ did not markedly modify these results. Of note these posterior means were in line with the ML estimate of the Recovery trial of 0.29, suggesting an increased relative weight of those data in the estimation process.

#### Handling heterogeneity through Pocock’s bias approach

Table 2 reports the posterior estimates of 28-day mortality when handling the alternate approach of Pocock, where differences were modelled by a normal parameter, with average bias either centered or not. Whichever the density of the bias, posterior mean estimates of 28-day mortality were all 0.2770, that is, close to those reached by fitting the prior to the historical data, though the width of credibility intervals were somewhat decreased.

**Fig. 5:**
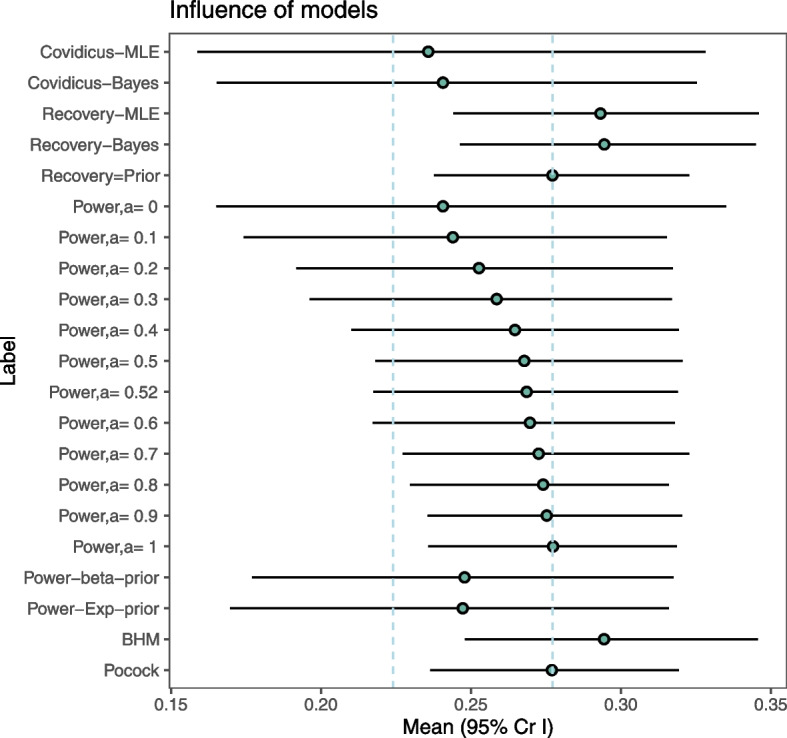
Estimation of 28-day mortality rate according to the handling of external data. MLE: maximum likelihood estimate. BHM: Bayesian hierarchical model. Pocock refers to the Pocock’s bias approach

As shown in Fig. 5 (and Table 2), the posterior estimate of 28-day mortality heavily depended on the assumption of the underlying heterogeneity between trials. However, the ability to quantify and compare the clinical differences of trials is crucial to determining applicability and use in clinical practice of results provided by sharing information across heterogeneous populations. Notably, apart from the statistical considerations, we considered the clinical implications of the decision to combine all doses in the analysis. Thus, given the potential heterogeneity in true benefit or harm from the two dexamethasone doses, we then aimed to compare the effect of the high- versus low-doses, based on the the data from the two (hypothetic) single arms. Nevertheless, as shown above in Table 1, there were obvious differences in treatment arms and patient populations, that could be handled. We thus wondered whether the 28-day mortality rate could differ across these datasets due to different dosages, that is, whether the treatment dosage could have influenced the outcome, considering the observed heterogeneity in trial populations.

### Comparing the 28-day mortality rate in response to low dose versus high dose of dexamethasone

**Fig. 6:**
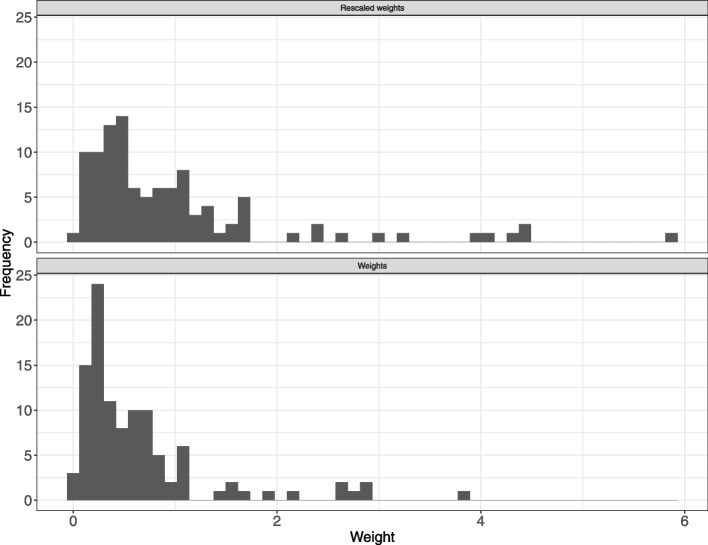
Distribution of weights

Based on published results from the Recovery trial on July 17, 2020, and individual data from the Covidicus trial, we first estimated a propensity score including age, sex, and comorbidity, as they appeared to be the main potential confounders in this setting. Weight distribution is shown in Fig. 6. There were few participants allocated near-zero weights, which would have indicated that the trials were quite different and may increase the uncertainty of the results. The mean weight was 0.7362, which increased to 1.00 after rescaling. After weighting, as expected, the characteristics of the weighted high dose group (Covidicus) reached those of the low doses group (Recovery), with a mean age of 58.8, 72% males, and 49% of patients with comorbidities (Table 3). The final step was to calculate the weighted outcome. The odds ratio for 28-day mortality was then estimated, with estimates ranging from 0.744 based on the original data down to 0.413 on the weighted data. In Table 4, the estimated weighted and unweighted estimates for the OR of 28-day mortality in the Covidicus trial to that in the Recovery trial, are shown. The weighted Covidicus results has shifted distant to Recovery; however, the 95% confidence interval increased. Thus, while the point estimate indicates that the difference between low- and high-dexamethasone arms might be higher when accounting for differences in age, sex, and comorbidities, the weighting procedure introduced some uncertainty, so conclusions drawn from the results should be carefully considered. Obviously, all of this relies on the underlying assumptions of no unobserved cross-trial differences, which may result in residual confounding, and of similar follow-up.

**Table 3:** Comparison of treatment groups in each trial, before and after weighting. SoC: Standard of Care; DXM: dexamethasone

Group	SoC	High DXM	High DXM weighted	Low DXM
Age, mean ± SD	65.2 ± 11.0	66.1 ± 10.7	58.8 ± 10.7	58.8 ± 11.3
Male sex, %	72.5%	80.4%	72.0%	72.0%
Comorbidities, %	74.3%	70.1%	49.0%	49.0%

**Table 4:** Estimated odds ratios (OR) with 95% confidence interval (95%CI) of 28-day mortality in the high-dose vs. low-dose dexamethasone treatment groups

Methods	OR (95%CI)
Unadjusted logistic regression	0.744 (0.448 to 1.237)
Weighted logistic regression	0.415 (0.204 to 0.839)
Bootstrap median OR	0.413 (0.215 to 0.697)

## Discussion

The use of historical data to empower inference in clinical trials where a control arm is either absent or contains insufficient data due to ethical issues, is of prime interest. To exemplify the main proposed statistical approaches to deal with external controls, we used a real-life illustration on the question of the benefit of corticosteroids in the COVID-19 patients, that was indeed a major challenge for clinicians at the first times of the COVID-19 outbreak. At that time, little data was available to describe the disease pathogenesis and treatment efficacy. Notably, the use of systemic corticosteroids was controversial [24], reported even to be possibly harmful in patients with COVID19-lung injury or shock [25].

We first aimed to borrow existing information to provide an estimate of the 28-day mortality rate, as the parameter of interest. Different approaches were considered that differ in their assumptions regarding the “closeness” of the parameter between the two sources of data. The simplest way was to directly use Bayes models, pooling all data, and ignoring data heterogeneity. It consists in eliciting some prior for the model parameter, then directly combined with the current data likelihood. Elicitation of the Beta prior parameters has been widely studied in the literature, and is growingly used in clinical trial design and analysis, as reported by Azzolina et al. in 2021 [26]. This may appear unrealistic in our setting given the observed differences between trials (Table 1). However, one may assume that such differences in patient features did not result in heterogeneity in treatment effect, as there were no consensus data at the time reporting predictive factors of treatment response in severe COVID-19 patients. Otherwise, the underlying assumption that the lowest dose will have a similar (even if not exact) effect as the highest dose (and vice versa) is often a fair assumption when pooling data, notably in the setting of systematic reviews. Moreover, such deviations in clinical heterogeneity of populations have been reported acceptable and might even increase the external validity of the pooled results [27].

Nevertheless, to allow the source data to be down-weighted to account for those differences between populations, notably in sample sizes, we first modified the Beta prior parameters, to decrease the shrinkage intensity. Other approaches could have been used. The prior could have been a weighted mixture of an informative prior and a vague component [6, 8, 28]. Otherwise, a prior predictive distribution derived from a meta-analysis of historical trials could be used [8].

**Fig. 7:**
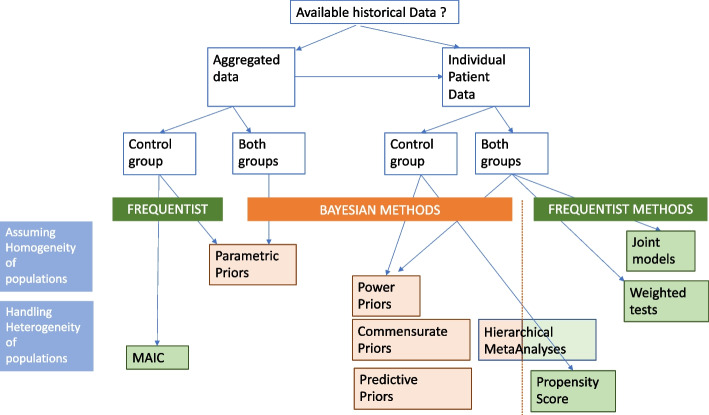
Summary of decision tools for handling external data

We then considered that the external data provided some biased mortality rates, either shifted [1] or rescaled using power models with an estimated power parameter. This inflates the variance of the historical prior (reducing the effective sample size of the historical data). However, the amount of discounting is subjective, with no easy operational interpretation. A meaningful range of the power parameter could be defined using some criterion, such the penalized likelihood-type criterion proposed by [29], though often leading to counter-intuitive results as reported by [30]. Thus, we preferred to use the empirical Bayes-type approach [18], that resulted in a down-weighting of about 50% of the historical sample size, erasing its potential influence. The results were slightly modified, with the mortality rate lying between estimates from the power models and those from the Bayesian model when ignoring heterogeneity in samples. This is line with previous results that heavily discounted the historical data unless it was very informative prior to the power prior. Otherwise, we considered both historical and current parameters to be exchangeable, with hierarchical (meta-analysis) models, where only variance reflects the heterogeneity across populations. Analysis revealed that posterior estimates heavily depended on the assumption regarding the closeness of the datasets. In such analyses, this also suggests the potential issue of data fishing, that is to choosing the estimate that is the closest to your own opinion. By contrast, we propose some guidance for defining the approach according to the assumptions regarding the closeness of past and current populations and data (Fig. 7). The use of power priors is an attractive and simple method to down-weight the influence of the historical data, thus taking into account some level of heterogeneity in populations and interventions. Moreover, the weight parameter is very interpretable for compatibility of studies and sample sizes when compared to the between study heterogeneity parameter in a hierarchical model. The difficulty in choosing the power parameter could be erased by using some criterion for decision-making such that proposed by [18]. This resulted in a posterior mean estimate of death rate of 0.2686 from our illustrative example, a likely compromise between separate estimates on each dataset. It is indeed based on the less constrained assumptions regarding heterogeneity in effects across trials, while down-weighting the influence of past data.

As a secondary objective, to compare mortality between historical and current trial data, we used MAIC which allows us to combine aggregated and individual patient data. Importantly, the targeted population was shifted to that of the Recovery trial, that is, based on younger patients with fewer comorbidities than in the Covidicus trial. This may explain the large difference observed in treatment effects compared to the naive group, with odds ratios ranging from 0.77 to 0.41, respectively. However, only differences in sex, age, and comorbidities were considered. Thus, one should take these results with caution, given indirect comparisons can be biased by both observed and unobserved cross-trial differences, which may result in residual confounding. Moreover, the ability to adjust for multiple baseline factors depends on having a sufficient number of patients and/or events in trials with IPD. Moreover, unlike traditional PS weighting, the availability of aggregate data for some trials in a MAIC prevents the use of existing methods for determining the fit and calibration of the PS model.

The risk in borrowing information from previous studies comes from borrowing from trials dissimilar to the concurrent trial in terms of prognostic factors and response to treatment, which may result in misleading the inference and thereby penalized estimates of the treatment effect [14]. Evaluating the similarity of the external control population to the clinical trial population is a multifaceted exercise. Violation of those assumptions can have drastic consequences for power and type I error if the historical information is biased.

Other methods could have been used. Commensurate priors [4] incorporate historical data but are adaptively robust to prior information that reveals itself to be inconsistent with the accumulated experimental data. Neuenschwander et al. [31] broaden the commensurate prior notion of historical data borrowing to the use of any trial-external complementary data (“co-data”), including both control and treatment data, and from trials that are either completed or ongoing. Otherwise, methods that estimate parameters for the between-trial heterogeneity generally offer the best trade-off of power, precision and type I error, with the meta-analytic-predictive prior being the most promising method [32].

Last, we exemplified available approaches to estimate the effect of dexamethasone in severely ill COVID-19 patients, ignoring the control arm of both trials. This was mostly justified by the desire to leave the estimates of treatment effect blinded at the time the analyses were planned; this further allowed us to obtain insights into easily understandable beta-binomial models. Of note, using comparative trial data is preferable whenever possible. All the approaches presented above could be easily applied to model some absolute or relative treatment effect rather than of the outcome.

## Conclusions

The potential role of observational studies in contributing to the body of evidence demonstrating drug and biologic product efficacy is important. A healthy degree of scepticism on the use of synthetic controls is thus expected from the scientific community [2]. The goal is to develop a path for ensuring that RWE solutions are an integral part of drug development and the regulatory life cycle at the FDA. Notably, it should be kept in mind that all methods are complex, and sensitive to parameter settings. These choices primarily depend on the available data and underlying assumptions. Accessing IPD appears mandatory to better handle potential bias by indication. None of those approaches appear robust enough to overcome RCT data.

## Data Availability

The datasets generated during and analyzed during the current study from the Covidicus trial are not publicly available due to French regulation, but are available from the corresponding author on reasonable request. The aggregated data from the Recovery trial were obtained from the published trial.
